# Genetic Engineering Strategies for *Euglena gracilis* and Its Industrial Contribution to Sustainable Development Goals: A Review

**DOI:** 10.3389/fbioe.2020.00790

**Published:** 2020-07-14

**Authors:** Ryo Harada, Toshihisa Nomura, Koji Yamada, Keiichi Mochida, Kengo Suzuki

**Affiliations:** ^1^RIKEN Baton Zone Program, Yokohama, Japan; ^2^RIKEN Center for Sustainable Resource Science, Yokohama, Japan; ^3^Euglena Co Ltd, Tokyo, Japan

**Keywords:** *Euglena gracilis*, SDGs, bioeconomy, genetic engineering, CRISPR-Cas9

## Abstract

The sustainable development goals (SDGs) adopted at the 2015 United Nations Summit are globally applicable goals designed to help countries realize a sustainable future. To achieve these SDGs, it is necessary to utilize renewable biological resources. In recent years, bioeconomy has been an attractive concept for achieving the SDGs. Microalgae are one of the biological resources that show promise in realizing the “5F”s (food, fiber, feed, fertilizer, and fuel). Among the microalgae, *Euglena gracilis* has the potential for achieving the “5F”s strategy owing to its unique features, such as production of paramylon, that are lacking in other microalgae. *E. gracilis* has already been produced on an industrial scale for use as an ingredient in functional foods and cosmetics. In recent years, genetic engineering methods for breeding *E. gracilis* have been researched and developed to achieve higher yields. In this article, we summarize how microalgae contribute toward achieving the SDGs. We focus on the contribution of *E. gracilis* to the bioeconomy, including its advantages in industrial use as well as its unique characteristics. In addition, we review genetic engineering-related research trends centered on *E. gracilis*, including a complete nuclear genome determination project, genome editing technology using the CRISPR-Cas9 system, and the development of a screening method for selecting useful strains. In particular, genome editing in *E. gracilis* could be a breakthrough for molecular breeding of industrially useful strains because of its high efficiency.

## Sustainable Development Goals (SDGs) and the Bioeconomy

The SDGs have taken over the millennium development goals (MDGs) established in 2001, and have been listed in the “2030 Agenda for Sustainable Development” adopted at the United Nations Summit in September 2015. The SDGs are international goals, which consist of 17 interconnected goals and 169 targets (United Nations, 2016) for the period from 2016 to 2030. These universal goals apply to all countries and aim at issues, such as inequality, sustainable consumption and production, and climate change, that need to be addressed, especially by developed countries. As the SDGs are designed to solve problems at a global level, there is the need for not only governments, but also private companies to work together (Scheyvens et al., 2016).

Bioeconomy is a concept to utilize materials, chemicals, and energy derived from renewable biological resources and is therefore closely associated with the SDGs (McCormick and Kautto, 2013). Hence, the expansion of the bioeconomy will contribute to the achievement of the SDGs. The European government has led several movements to popularize bioeconomy since the mid-2000s (McCormick and Kautto, 2013). Achieving the bioeconomic vision requires the active participation of the general public and the commitment of governments and industries to drive concerted efforts on the sustainable development of the bioeconomy (McCormick and Kautto, 2013).

## Contribution of Microalgae to the SDGs

Microalgae are organisms that grow in a variety of environments, including freshwater, seawater, wet soils, and rocks and form the basis of aqueous food chains (Dittami et al., 2017). They are known to produce useful physiologically active compounds for use as functional foods as well as lipids for use in biofuels. Therefore, they are considered to have a potential to realize the goals of the bioeconomy. In many countries, microalga-related developments require substantial investments and have successfully progressed from the research phase to the demonstration phase (Su et al., 2017). The major commercial areas where microalgae can be used are food (Christaki et al., 2011), fuel (Mofijur et al., 2019), cosmetics (Ariede et al., 2017), healthcare (Nethravathy et al., 2019), and feed (Dineshbabu et al., 2019). In addition, microalgae show promise in their application in wastewater treatment (De Francisci et al., 2018) and the reduction of carbon dioxide (CO_2_) emissions (Klinthong et al., 2015). Utilization of algae for wastewater treatment and CO_2_ emission reduction contributes to the “SDG-6: ensure availability and sustainable management of water and sanitation for all” and the “SDG-13: take urgent action to combat climate change and its impacts.”

## Industrial Uses of *E. gracilis*

### Growth Advantages of *E. gracilis* Compared to Other Euglena Species

The microalga *E. gracilis* has been used as a model organism in basic research for decades, especially to study photosynthesis. Besides *E. gracilis*, the genus *Euglena* includes more than 200 species, some of which have beneficial characteristics, such as the production of paramylon and several other biologically active substances, and are thus valuable for industrial use. Among these species, *E. gracilis* shows a much faster growth rate than other *Euglena* species under optimal conditions (Suzuki et al., 2015), making it more suited for mass culture and commercialization. Microalgae are known for their ability to take up toxic heavy metals from the environment, leading to the induction of a heavy-metal stress response in them (El-Esawi, 2019). Similar to other microalgae, *E. gracilis* is resistant to the stress caused by heavy metals such as Cd^2+^, Cr^3+^, Hg^2+^, Cr^6+^, Pb^2+^, UO${}_{2}^{2+}$, and Zn^2+^ (El-Esawi, 2019) and has good physical and metabolic adaptability (Rodríguez-Zavala et al., 2007; Lira-Silva et al., 2011; García-García et al., 2018; El-Esawi, 2019). This finding indicates that wastewater can be used to culture *E. gracilis* even on uncultivated land.

### Characteristics of Paramylon Obtained From *E. gracilis*

Paramylon is a β-1,3-glucan synthesized by *E. gracilis*, which accumulates in the cells. These glucan crystals can be used in a wide range of industrial applications and be degraded to wax esters under anaerobic conditions. β-1,3-glucan is a polysaccharide abundant in fungi including mushrooms and is widely recognized as a useful component owing to its immune boosting properties (Nakashima et al., 2018b). Paramylon from *E. gracilis* has a similar effect and its role as a functional food has been long studied (Nakashima et al., 2018b; Barsanti and Gualtieri, 2019). The paramylon content in *E. gracilis* Z can be as high as 60–70% of the dry weight of the cell (Yasuda et al., 2018).

### Characteristics of Wax Esters From *E. gracilis*

*Euglena gracilis* is capable of fermenting paramylon under anaerobic conditions and producing large amounts of wax esters (Inui et al., 1982; Zimorski et al., 2017). This reaction involves the anaerobic breakdown of paramylon into glucose units and an enzymatic metabolism to pyruvate, which is then oxidized by the O_2_-sensitive enzyme pyruvate:NADP+ oxidoreductase to produce acetyl-CoA in the mitochondria (Rotte et al., 2001). Acetyl-CoA functions as the C2 donor in the reverse β-oxidation reaction to form acylated CoA (Inui et al., 1984). Next, acyl-CoA is exported to the endoplasmic reticulum, reduced to fatty alcohols, and esterified with another molecule of acyl-CoA to form a wax ester (Teerawanichpan and Qiu, 2010). This series of anaerobic processes constitute the wax ester fermentation. As wax esters can be commercialized as raw materials for biofuels, many genetic studies have focused on the analysis of metabolic pathways with the aim of increasing their production (Inui et al., 2017). The analysis of sulfur metabolites using LC/MS has shown that degradation of glutathione and proteins occurs as a secondary reaction during wax ester fermentation (Yamada et al., 2019). The secondary reaction in *E. gracilis* generates hydrogen sulfide, which is empirically known to be accompanied by wax ester fermentation reactions.

### Advantages of the Industrial Use of *E. gracilis* for “5F” Products

**FIGURE 1:**
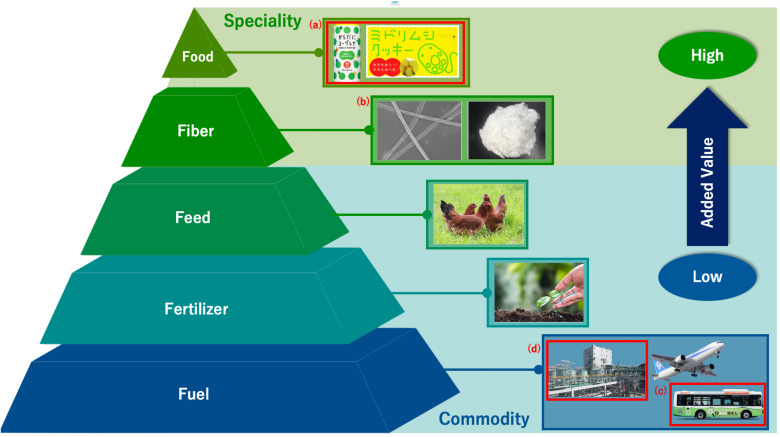
Concept of 5F of biomass using *E. gracilis*. Each product is arranged from the top in the order of added value. Those marked in red are already in operation or commercialized. **(A)**: Foods are drink and cookie they include *E. gracilis.* **(B)**: Fiber include rayon which contains paramylon (Euglena Co Ltd and Omikenshi Co Ltd, 2020). **(C)**: Fuel derives from *E. gracilis* has already used for bio diesel. **(D)**: There is manufacturing plant for bio jet fuel and diesel fuel derives from *E. gracilis* in Japan.

The use of *E. gracilis* in industry is expected to grow further due to the establishment of a mass culture technology. This large-scale commercial cultivation of *E. gracilis* began in 2007 after a successful mass cultivation in 2005, which focused on improving its yield and drying processes (Suzuki, 2017). Since then, *E. gracilis* has been actively studied to enhance further commercialization. Commercialization with *E. gracilis* is based on the strategy known as the “5F of biomass” or “5F”s, which represent food, fiber, feed, fertilizer, and fuel (Suzuki, 2017) (Figure 1). The “5F”s of biomass indicate biomass utilization listed in a descending order of added value; the products at the higher level are relatively more expensive than those positioned at the lower level. For example, “food” is located at the top of this list owing to its high unit price, which can be further increased using marketing- and supply strategies. On the other hand, the other “F”s, namely, feed, fertilizer, and fuel are of comparatively less value per unit in the commodity area. For example, price per weight of petroleum is low and is a competitor of biomass fuel. Therefore, it is necessary that biomass fuel be supplied at low cost. Food products derived from *E. gracilis*, ranking highest on the list of “5F”s, have already met with success (Suzuki, 2017), and the other “F”s will soon follow suit.

A variety of nutritional products for human and animal consumption are obtained from *E. gracilis*, leading to its commercial use as a food ingredient. Commercialization of *E. gracilis* as food is an established practice undertaken by many organizations in several countries including Japan and the United States (Gissibl et al., 2019). Studies evaluating the nutritional value of microalgae have been conducted for over 50 years, and these values have been well documented. *Chlorella* species are rich in amino acids, fatty acids, and vitamins (Morimura and Tamiya, 1954). The accurate analysis of cell components for each culture condition conducted using the *E. gracilis* Z strain (Kitaoka and Hosoya, 1977) revealed that *E. gracilis* has high levels of the amino acid, methionine, which is comparable to casein in terms of nutritional value, and is a rich source of proteins (Table 1) (Kitaoka and Hosoya, 1977). Although *E. gracilis* requires vitamins B1 and B12 as essential nutrients for growth, it is known to synthesize many of the other vitamins (Baker et al., 1981). The production of vitamins C and E can be increased by modifying the culture conditions (Table 1; Kitaoka, 1989). The fatty acids synthesized by *E. gracilis* are primarily highly unsaturated and their composition varies significantly depending on the culture conditions. When *E. gracilis* is grown under illumination, there is an increase in the unsaturated fatty acids including C16 and C18; under dark conditions, palmitic acid, abundant in phospholipids and long-chain fatty acids C20 and C21, is the primary product (Hulanicka et al., 1964).

**TABLE 1:** Compositions of *E. gracilis* Z (Based on Kitaoka and Hosoya, 1977).

		GL**	GD**	**Autotrophic condition**
General composition of *E. gracilis* Z*	Clude proteins	54.81	31.75	58.71
	True proteins	45.97	25.53	50.94
	Clude fats	15.19	8.71	15.5
	Ashes	4.96	2.13	6.8
	Paramylon	17.6	53.77	15.97
	Residues	7.44	3.64	3.02
	Water	3.49	2.54	6.81
Composition of essential amino acids† and amino acid values	Isoleucine	3.3	3.3	3.5
	Leucine	6.5	6.5	7.3
	Lysine	5.9	5.8	5.6
	Sulfur-containing amino acids	4.4	4.4	3.2
	Aromatic amino acids	7.2	7.2	7.6
	Threonine	4.1	4.4	4.3
	Tryptophan	1	1	1.1
	Valine	4.7	5.3	5
	Amino acid value	83	83	88
Composittions of vitamins in *E. gracilis* (mg/100 g and ‡ug/100 g)	Vitamin B1 ‡^a^	27.9	25.3	1
	Vitamin B2	5.6	4.3	4.5
	Vitamin B6	1.07		
	Vitamin B12 ‡^a^	10.1	30.5	36.6
	Nicotinic acid and Nicotinamide	37	41.4	28.3
	Biotin‡	79	88	14
	Pantothenic acid	3.4	2.9	3.6
	p-aminobenzoic acid			63
	Ascorbic acid	425	61	345
	Carotene	174	9.4	10.5
	Vitamin E	155	31	
	Vitamin K1		0.5	

*Freeze-dried sample, Except for water, values indicate parcentage of compositions per dry weight. **GL: Wild strain cultured under light condition, GD: Wild strain cultured under dark condition. ^†^Grams of amino acids per 16 g of nitrogen. ^*a*^Vitamin is added in the culture medium. ‡ug/100g.

Unlike other microalgae, *E. gracilis* lacks a cell wall, and is therefore easy to be digested when consumed. This is a desirable feature in nutritional supplements. Commercially, *E. gracilis* is made available as a dry powder intended to be added to beverages or to bakery products such as cookies. It is also sold as a supplement by Euglena Co,. Ltd., (Japan) in the form of capsules. *E. gracilis* can therefore be used as a nutritional supplement in various foods. In addition, Euglena Co., Ltd., obtained Halal certification for *E. gracilis* in 2013; therefore, a high demand for this microalga as a nutritional substitute for animal protein is anticipated from the vegetarian population and religious communities that follow Halal practices. As *E. gracilis* does not require agricultural land for production, the large-scale production does not compete with the production of crops, which will contribute to the remediation of the food problem. In developed nations, *E. gracilis* is in demand as an ingredient in functional foods depending on the function of paramylon. Studies on the immunomodulatory functions of paramylon have reported an amelioration of influenza symptoms (Nakashima et al., 2017), atopic dermatitis (Sugiyama et al., 2010), and rheumatic disorders (Suzuki et al., 2018). Furthermore, oral administration of paramylon inhibits hyperglycemia in type 2 diabetic OLETF rats (Shimada et al., 2016) and exhibits antifibrotic activity in NASH mice (Nakashima et al., 2018a). Paramylon also displayed anti-obesity and anti-inflammatory effects in mice fed a combination of *E. gracilis* and vegetables (Okouchi et al., 2019).

Paramylon is considered to be an alternative for petroleum-based plastics and a natural compound that does not compete with other petroleum products. The production of plastics is accompanied by a large emission of greenhouse gases and a consumption of enormous amounts of energy. Therefore, the use of biomaterials in the manufacture of plastics is an environmentally friendly approach. A study shows that paramylon functions as a filler in bioplastics and improves the final product by causing an increase in the maximum stress point and elasticity, and by decreasing the maximum displacement point (Suzuki et al., 2013). A plastic material composed of paramylon and the fats and oils derived from the shells of cashews has been developed. This material shows higher heat resistance than do traditional bioplastics such as polylactic acid and nylon 11 (AIST, 2013). Under anaerobic conditions, *E. gracilis* produces succinic and lactic acids, which can be used as ingredients for the manufacture of plastics (Tomita et al., 2016). Therefore, the *E. gracilis* biomass can be used in the manufacture of bioplastics owing to its environmentally friendly properties.

The role of *E. gracilis* in enhancing the properties of feed has also been studied. The addition of paramylon to the culture environment during the cultivation of *Artemia*, a raw fish feed, imparts a stress resistance property to this genus (Vismara et al., 2004). Moreover, *E. gracilis* biomass is being considered as a functional feed for livestock to reduce methane generation. Studies suggest that methane emissions from ruminant livestock, due to the presence of large amounts of methanogens in the rumen, have a major impact on global warming (Johnson and Johnson, 1995). It has also been reported that the addition of soybean oil to the feed significantly reduces the presence of methanogens in the rumen, which results in reduced methane emissions (Lillis et al., 2011). The wax esters of *E. gracilis* are rich in medium chain fatty acids, primarily myristic acid, which when mixed with feed might be responsible for the significant reduction of methane emissions (Aemiro et al., 2018). Moreover, hay mixed with *E. gracilis* has been shown to increase the nutritional value of feed for sheep (Aemiro et al., 2017), indicating the potential of *E. gracilis* as feed in livestock.

Microalgae including *E. gracilis* are of great value as fertilizers and can help increase agricultural output. High yields of crops are currently achieved using pesticides and inorganic fertilizers that make the sustainable use of arable land practically impossible (Vox et al., 2010). Therefore, an organic fertilizer that is safe and induces high crop yields is necessary. The microalgae that have been studied for use as fertilizers include *Acutodesmus dimorphus* (Garcia-Gonzalez and Sommerfeld, 2015), *Chlorella spp.*, *Neochloris conjuncta*, *Botryococcus braunii* (Uysal et al., 2016), and *Nannochloropsis oculata* (Coppens et al., 2015). Microalgae are mainly used as fertilizers to supply nitrogen (Uysal et al., 2016). When used as a fertilizer, *N. oculata* improves the sugar and carotenoid contents of tomatoes, but decreases the crop yield (Coppens et al., 2015). It has been reported that paramylon produced by *E. gracilis* functions as a bio-stimulant in plants (Levine et al., 2014). Moreover, the use of fertilizers containing *E. gracilis* and paramylon stimulates growth, improves abiotic stress tolerance, and improves crop yield, thereby indicating *E. gracilis* potential as an adjuvant to fertilizer use (Levine et al., 2014).

The wax ester accumulated in *E. gracilis* is mainly composed of myristyl myristate (C28), which is categorized as a third-generation biofuel, indicating that *E. gracilis* may be appropriate for use as a biofuel (Inui et al., 2017). Fatty acid methyl ester and the hydrocracked alkane of the wax have lower freezing points than other microalgal biofuels that are rich in medium chain fatty acids such as palmitic- and stearic acids (Hu et al., 2008), and are therefore suitable as biojet fuels (Klopfenstein, 1985). In addition, wax esters have been shown to produce larger amounts of paraffins and olefins instead of flame-retardant aromatics than catalytic cracking of triacylglycerols. This indicates that the lipids from *E. gracilis* are suitable ingredients for biofuel production using catalytic cracking (Shimada et al., 2018).

## Research Challenges Involved in the Genetic Modification of *E. gracilis*

Unlike other microalgae, the presence of paramylon and wax esters in *E. gracilis* imparts properties conducive to its industrial use. The auxotrophy of vitamins B1 and B12 is believed to be responsible for their limited yields by *E. gracilis*, even after technical optimization of cultivation techniques (Gissibl et al., 2019). Therefore, supplementation of these vitamins by scaling up cultivation, co-culturing *E. gracilis* and bacteria that produce the required nutrients, and production of transgenic *E. gracilis*, are a few solutions to overcome this problem (Gissibl et al., 2019). Co-culturing microalgae and microalga growth-promoting bacteria (MGPB) is known to increase biomass production (Fuentes et al., 2016; Ramanan et al., 2016; Toyama et al., 2019) *Emticicia sp.* EG3 was the first MGPB to be discovered and used in co-culture with *E. gracilis*, which enhanced the biomass production more than three-fold (Toyama et al., 2019).

Although co-culture with MGPB may be a solution for improving the yield of *E. gracilis*, breeding the strain is a more straightforward procedure to achieve high yields of the desired products. To achieve effective breeding aimed at improving the production of *E. gracilis* to suit individual purposes, technologies targeting classical mutant screening and targeted gene modification are needed, and some of these techniques have been developed. Using classical mutant screening, methods to effectively induce mutations in *E. gracilis* have been explored. In the next section, the history and prospects of technology development concerning gene regulation and breeding of *E. gracilis* are described.

### Complete Nuclear Genome Sequencing

The complete genomes of chloroplast and mitochondria of *E. gracilis* were determined in 1993 and 2015 (Hallick et al., 1993; Dobáková et al., 2015), respectively. However, the complete nuclear genome sequence of *E. gracilis* has not been determined and only a draft genome is available (Ebenezer et al., 2017). The nuclear genome of *E. gracilis* is 1.4–2.0 Gbp (Ebenezer et al., 2017), which is approximately 10–50 times larger than that of other algae, and estimated to contain repetitive sequences at a high frequency. Therefore, it is necessary to obtain not only the short reads, but also the long read sequences using a sequencer (Ebenezer et al., 2017). There is also a need for new pipelines to be developed in bioinformatics to address the high ploidy of the nuclear genome of *E. gracilis* (Ebenezer et al., 2019).

For a large nuclear genome containing a high frequency of repetitive sequences, it may be useful to apply the techniques used to determine plant genomes. Plants amplify their genomes using transposon amplification during the process of evolution and domestication. Repetitive sequences, transposable elements, and gene duplication in particular have significantly increased in gymnosperm genome (Mackay et al., 2012). The similarity of these paralogs creates ambiguity during genome assembly using short-read next-generation sequencers. These ambiguities make it difficult to construct a complete genome and some sequences may be lost. In addition, short-read mapping requires a lot of computer resources. One way to solve these problems is to generate long reads beyond the region of the repetitive sequence and provide them to ambiguous regions. Long read sequences are provided by Sequencing Platforms such as TruSeq Synthetic Long Read (Illumina), SMRT (PacBio), and ONT (Oxford Nanopore), among which, the PacBio SMRT sequencer is most widely used. *De novo* assembly by PacBio is more continuous and complete than other approaches. The nuclear genome of *E. gracilis* is difficult to study owing to its large size, repetition, and complexity (Gumińska et al., 2018). Therefore, it is necessary to obtain high-quality DNA samples from the absorbance ratios point of view for enough data to provide adequate coverage to enable genome assembly (Gumińska et al., 2018). CTAB-based DNA purification methods are more efficient and accurate than commercial kits and methods using phenol-chloroform extraction (Gumińska et al., 2018).

### Transient Gene Silencing Using RNA Interference

RNAi is a frequently tool used for transient gene regulation in several microorganisms including *E. gracilis*. This technique was first established in nematodes and trypanosomes (Fire et al., 1998; Ngô et al., 1998) and then applied to *E. gracilis* to regulate photoactivated adenylyl cyclase (Iseki et al., 2002). Knockdown of genes by RNAi using double-stranded RNA is only a transient method to elucidate gene function and therefore, cannot be used to breed useful strains. However, RNAi has been an effective tool in knocking down genes such as redox-related enzymes in *E. gracilis* (Ozasa et al., 2017). RNAi has also been used in the knockdown of *EgSTD1* and *EgSTD2*, which are related to the wax ester fermentation pathway (Kimura and Ishikawa, 2018).

### Transgene Introduction Techniques

Several transgenic technologies like particle gun, electroporation, and *Agrobacterium*-mediated transformation have been reported to introduce transgenes into *E. gracilis* cells (Ohmachi et al., 2016; Shigeoka et al., 2016; Khatiwada et al., 2019). Stable transgenic lines are claimed to have been produced by introducing a linear DNA gene expression cassette derived from T-DNA using the particle gun method (Shigeoka et al., 2016). Transgene introduction techniques have been used to study photosynthesis with the aim of enhancing wax ester production. Consequently, the transgenic *E. gracilis* (EpFS) line, which expresses fructose-1,6-bisphosphatase and sedoheptulose-1,7-bisphosphatase involved in the cyanobacterial Calvin cycle, has been created (Ogawa et al., 2015). Comparison of the photosynthetic capacity and wax ester productivity of the transgenic strain with that of the wild type showed that photosynthetic activity was significantly higher and the wax ester production was 13–100 times higher in the transgenic strain (Ogawa et al., 2015). Therefore, gene transfer may be a useful tool for artificial regulation of metabolic pathways. However, the culture of genetically modified *E. gracilis* may be prohibited by local laws pertaining to genetically modified organisms (GMOs) (Behera, 2014). Furthermore, the long-term stability of the introduced transgenes has not been evaluated, suggesting that the industrial use of these transgenic lines could possibly give rise to unforeseen problems such as impacts on the human body and environment. Therefore, there is a need for reliable methods with better stability.

### Classical Breeding Methods for Creating Nuclear Mutants

Using nuclear mutant organisms other than GMOs for industry is not regulated by law in many countries. Therefore, various forward genetic approaches have been attempted to create nuclear mutants of *E. gracilis*. These approaches are described as classical breeding because they depend on the expression of new traits upon spontaneous mutations. Traditionally, UV and X-rays as physical mutagens, and NTG and EMS as chemical mutagens are used to produce *E. gracilis* mutants. However, many such mutants are thought to be derived from mutations in the chloroplast genome (Schiff et al., 1980). The production of *E. gracilis* mutants deriving its phenotype from the mutations in the nuclear genome is thought to be difficult due to its genomic redundancy or the highly efficient DNA repair mechanisms (Yamada et al., 2016a). Introduction of mutations using heavy ion beams efficiently induces mutations in higher plants (Kazama et al., 2013). Although the underlying mechanism is not fully understood, high-temperature tolerant mutants and lipid-rich mutants, whose phenotypes are probably derived from mutations of the nuclear genome, have been successfully produced by irradiating *E. gracilis* using a heavy ion beam (Yamada et al., 2016a, b).

### Methods for Selecting Useful Strains

Mutants with useful traits need to be chosen for selective breeding. Selection of mutants of *E. gracilis* requires devising and experimentation with classical screening methods, such as live- and a behavior-based selections. This is of particular importance in the commercial application of biofuel made from lipids obtained from *E. gracilis*, which necessitates the breeding of *E. gracilis* strains that can produce more wax esters. A study reported an improved fluorescence-activated cell sorting-based selection method for *E. gracilis* wherein BODIPY staining of the cellular lipids and large nozzles were used to sort live cells to obtain such mutants (Yamada et al., 2016a). Using this method, a strain containing 40% more lipids than the wild type was segregated from the Fe-ion-beam irradiated mutant population (Yamada et al., 2016a). In addition, a selection method using a microfluidic device has been successful in classifying *E. gracilis* based on the shape of individual cells (Li C. et al., 2017; Li M. et al., 2017). This method is expected to become a powerful screening technique when extended to and integrated with metabolic and genetic engineering technologies. In addition to these sorting techniques, non-staining imaging techniques for lipids and polysaccharides in live individual *E. gracilis* cells have been developed using Raman micro-spectroscopy, a technique helpful in determining the unique molecular information of *E. gracilis* by analyzing the wax ester fermentation (Iwasaki et al., 2019). At present, large-scale label-free single-cell analysis of paramylon in *E. gracilis* is possible (Hiramatsu et al., 2020).

### Genome Editing of *E. gracilis* Using CRISPR-Cas9

**FIGURE 2:**
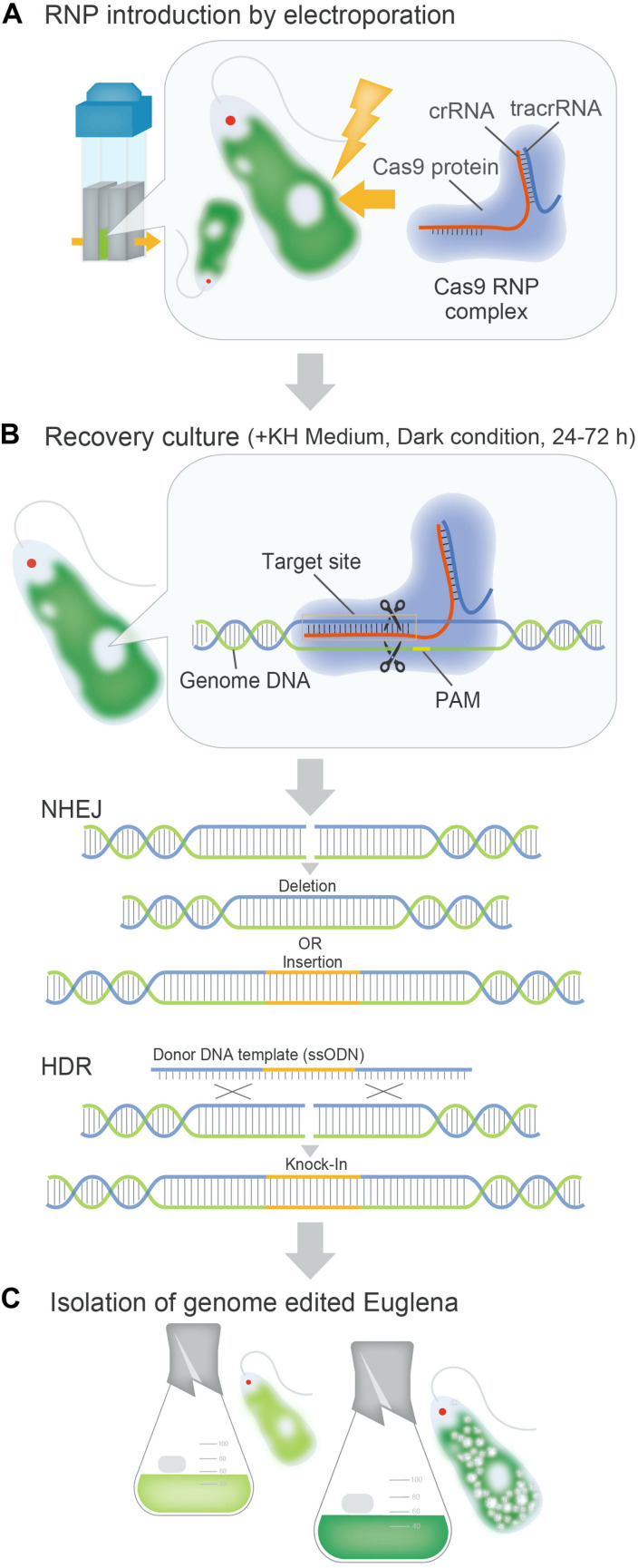
Overview of the Cas9 RNP-based genome editing process in *E. gracilis*. **(A)**: Direct delivery of Cas9RNP complexes to *E. gracilis* cells by electroporation. **(B)**: Mutagenesis events during recovery culture after introduction of Cas9 RNPs into *E. gracilis* cells. **(C)**: Establishment and industrial use of functionally modified euglena strains.

The utilization of genome editing in *E. gracilis* could be a breakthrough for molecular breeding of industrially useful strains. Strains created using the genome editing technology employing the ribonucleoprotein (RNP) complex are transgene-free and are suitable for industrial use. At present, RNP-based genome editing of microalgae using CRISPR-Cas9 (Cong and Zhang, 2014) has been actively performed in various species, but many selection-free methods display a low mutation efficiency (∼1%) (Table 2; Baek et al., 2016; Shin et al., 2016; Ferenczi et al., 2017; Serif et al., 2018; Yoshimitsu et al., 2018; Findinier et al., 2019; Naduthodi et al., 2019; Nomura et al., 2019). In *E. gracilis*, a highly efficient genome editing method was recently established based on a Cas9-RNP introduction procedure, which does not depend on the expression of the transgene in the cell (Figure 2). In the first case, the Cas9 RNP targeting the gene *EgGSL2* involved in the biosynthesis of paramylon in *E. gracilis* (Tanaka et al., 2017) was introduced using electroporation. Direct delivery of Cas9 RNPs into the cell achieved a non-homologous end joining-mediated indel mutation rate of up to 90.1% (Nomura et al., 2019). This rate is much higher than the mutation efficiency of Chlamydomonas (∼10%) and other microalgae (∼1%) when using the RNP-based genome editing method (Jeon et al., 2017; Spicer and Molnar, 2018). In addition, using single-stranded oligodeoxynucleotide as donor DNA template, precise knock-in (∼70%) of 42 bp exogenous DNA sequences via homology-induced repair has been successfully achieved (Nomura et al., 2019). In many microalgae, except *E. gracilis*, the lower efficiency of genome editing is related to the presence of cell walls or structures on the cell surface (Jeon et al., 2017). It is speculated that the introduction of RNP is less inhibited in *E. gracilis* than in other microalgae, due to the lack of a cell wall in the former. The high efficiency of genome editing that cannot be achieved in other microalgae leads to the stable mutagenesis or the transformation of *E. gracilis*. This is one of the advantages of the industrial use of *E. gracilis* as a controllable microalga.

**TABLE 2:** Examples of RNP-based genome editing of microalgae.

**Species**	**RNP type**	**Delivery**	**Exogenous DNA**	**Target gene**	**Selection**	**Efficiency**	**References**
*Chlamydomonas reinhardtii* CC-124	SpCas9	Electroporation	Free, linearized Plasmid	*MAA7, CpSRP43, ChlM*	5-FI, Hygromycin and Clony color	0.17–40%	Shin et al., 2016
*Chlamydomonas reinhardtii* CC-4349	SpCas9	Electroporation	Free	*CpFTSY, ZEP*	Free	0.007–1.11%	Baek et al., 2016
*Chlamydomonas reinhardtii* CC-1883	LbCpf-1 (LbCas12a)	Electroporation	ssODN	*FKB12, CpFTSY, CpSRP43, PHT7*	Rapamycin, Clony color or size	0.5–16% (Scarless 0.1–10%)	Ferenczi et al., 2017
*Coccomyxa* sp. strain KJ	SpCas9	Electroporation	Free	*KJFTSY*	Free	0.01%	Yoshimitsu et al., 2018
*Phaeodactylum tricornutum*	SpCas9	Particle bombardment	Free	*PtAureo1a* with *PtUMPS, PtAPT*	5-FOA, 2-FA	65–100%	Serif et al., 2018
*Nannochloropsis oceanica* IMET1	SpCas9, FnCas12a, LbCas12a, AsCas12a	Electroporation	dsDNA	*NR*	Zeocin	0–93%	Naduthodi et al., 2019
Chlamydomonas reinhardtii CC-4533	SpCas9	Electroporation	dsDNA	*CrFzl*	Paromomycin	47.5%	Findinier et al., 2019
*Euglena gracilis*	SpCas9	Electroporation	Free	*EgGSL2*	Free	77.7–90.1%	Nomura et al., 2019

## Conclusion

*Euglena gracilis* can produce commercially useful substances, such as paramylon and wax esters, that cannot be biosynthesized by other microalgae. These products find use in several applications and contribute to the “5F of biomass.” The commercial profitability of *E. gracilis* in the food industry has already been demonstrated. Prior to further industrial use of *E. gracilis*, it is necessary to control its production using genetic technology, especially complete nuclear genome sequencing and efficient gene technology. Complete nuclear genome sequencing has not yet been achieved and requires the development of efficient pipelines for long read acquisition and the assembly of acquired genes. For the latter, CRISPR-Cas9 has established itself as an efficient mutagenesis technology for *E. gracilis* as compared to its application in other microalgae. Techniques for selecting useful strains are constantly being developed and evaluated. These technologies have the potential to provide knowledge on *E. gracilis* gene function and achieve efficient breeding. We can thus expect obtaining products and services from *E. gracilis* for people and improving the environment to achieve the SDGs.

## Author Contributions

RH wrote this manuscript with support from TN, KY, and KS. TN aided in writing this manuscript and created Figure 2. KY and KM aided in writing this manuscript. KS aided in writing this manuscript and supervised this work. All authors contributed to the article and approved the submitted version.

## Conflict of Interest

KY and KS are employees of Euglena Co Ltd, which is a private company selling *E. gracilis* products.

The remaining authors declare that the research was conducted in the absence of any commercial or financial relationships that could be construed as a potential conflict of interest.

## Abbreviations

SDGs: sustainable development goals
MDGs: millennium development goals.
